# Managing Cystic Fibrosis in Polish Healthcare

**DOI:** 10.3390/ijerph17207630

**Published:** 2020-10-20

**Authors:** Marta Rachel, Stanisław Topolewicz, Andrzej Śliwczyński, Sabina Galiniak

**Affiliations:** 1 Institute of Medical Sciences, Medical College, University of Rzeszów, 35-959 Rzeszów, Poland; sgaliniak@ur.edu.pl; 2 Department of Allergology and Cystic Fibrosis, State Hospital 2 in Rzeszow, 35-301 Rzeszów, Poland; 3 Mechanical School, Rzeszów 35-078, Poland; statop.414@edu.erzeszow.pl; 4 Division of Quality Services, Procedures and Medical Standards, Medical University in Łódź, 90-647 Łódź, Poland; andrzej.sliwczynski.ahe@gmail.com

**Keywords:** cystic fibrosis, deaths, drug program, hospital treatment

## Abstract

The quality and length of life of patients with cystic fibrosis (CF) are determined by a number of factors including the quality of healthcare received by patients, as well as access to drug programs dedicated to this particular disease. The purpose of this paper is to present an overview of changes in the average life expectancy and mortality rate of the CF population in Poland between 2000 and 2018. Furthermore, we would like to evaluate access to healthcare services, including the drug program, guaranteed by public healthcare system, and funded by National Health Fund (NHF). The average life expectancy of patients with CF increased in the period in question from ca. 14.5 ± 7.6–24.5 ± 8.9 years (mean ± SD, *p* = 0.0001). We have observed a drop in the number of deaths in paediatric age during that period. Despite the increase in life expectancy, the use of health resources in patients with CF, especially the drug program, is dramatically low. Considering the fact that in Poland there was no active countrywide CF registry, now it is possible to estimate the frequency of use of CF healthcare services in various provinces exclusively on the basis of database maintained by the Polish NHF.

## 1. Introduction

Cystic fibrosis or CF (dysporia broncho-entero-pancreatica congenita familiaris) is classified as a rare disease and is the most frequent monogenic disease in Caucasian patients inherited in a recessive autosomal manner. According to the current data obtained from newborn registries and screening programs, the incidence rate of CF in Europe is 0.737 per 10,000 births. Global statistics show that differences depend on the population and region, and range from 1:2838 in France, 1:1800 in Slovakia, and 1:2833 in Czechia to 1:3170 in Italy, 1:6000 in Portugal, and 1:4750 in the Netherlands. Cystic fibrosis is most often diagnosed in Ireland where the incidence rate is 1:1353, in Sweden with incidence rate ranging from 1:2200–1:5600, among the patients of Caucasian origin in North America (1:3608) and in Ashkenazi Jews (1:1800–1:4000). According to the literature data, the disease is decisively less frequent among patients of other races in Southern and Central America (1:12,163), Africa (1:13,924), or Asia (1:350,000) [1,2,3,4].

CF is a systemic disorder in which the quality and length of the patient’s life is usually determined by changes in the respiratory system, and the cause of death is in most cases respiratory failure. Life expectancy of patients with CF depends on a number of factors, including ethnic origin, lung function or chronic bacterial infections [5,6]. However, one of the key factors seems to be the quality of healthcare received by the patients; the other is access to drug programs dedicated to the disease. In many highly developed countries with publicly funded healthcare systems, CF has no longer been regarded as a paediatric disease for many years now; it is estimated that over 50% of the population of CF patients in such countries are adults, and the average age of the population has been growing steadily [7,8,9,10,11,12]. Along with the significant increase of the average life expectancy of CF patients, the clinical spectrum of this complex multi-systemic disease is constantly evolving [13]. This is the result of the development within many areas of CF care. Many factors are responsible for these major advances in treatment such as standardisation with high-quality healthcare of patients in specialised centres by multidisciplinary teams, better control of pulmonary infection with the development of new inhaled therapies, better control of *Pseudomonas aeruginosa* colonisation, availability of modern treatment including the cystic fibrosis transmembrane conductance regulator (CFTR) modulators, aggressive nutritional supplementation with pancreatic enzymes, early diagnosis through newborn screening, and lung transplantation [14,15,16]. In spite of this development, CF remains a progressive lethal disease. According to European CF treatment standards, the comprehensive healthcare system should include diagnosis, prevention of development and treatment of exacerbated forms of the bronchopulmonary disease, as well as monitoring, treatment of complications and comorbidities [17,18]. Nevertheless, it is clear that despite the same recommendations, the organisation and level of care for CF patients varies across Eastern Europe. In many countries, basic aspects of healthcare are missing, including staff training, continuous development programs, or access to therapies, which is especially important due to the increasing number of adult patients with CF [19,20]. It is vital to ensure provision of care in well-organised, multi-disciplinary CF treatment centres, with medical staff of appropriate expertise and effective drugs to treat the infection and improve the mucociliary clearance.

The research hypothesis was that CF patients take full advantage of the benefits, including the drug program, guaranteed by the public healthcare system and financed by the National Health Fund (NHF) in Poland during the 2008–2018 period and that translates into extension of their lifetime. Moreover, we would like to show how the average life expectancy and mortality rate of the CF population in Poland changed over time from 2000–2018.

## 2. Materials and Methods

The current study summarises the state of knowledge based on the data collected by the NHF in Poland, Demographic Database of the Polish Central Statistical Office, report on the current situation of CF patients [21], and CF treatment centres audit [22] related to services provided to CF patients in individual provinces (voivodeships) in Poland, the use of the drug program, and the number of deaths due to CF.

For the purpose of this analysis, the National Health Fund’s (NHF) reporting databases were used to obtain details of patients for the 2008–2018 period. Poland has both public and private healthcare systems. CF patients remain mainly under the care of the public health service due to multidisciplinarity and high cost of the treatment. The procedures and treatment performed are reported to the regional branches of the NHF, and then to the NHF’s central office. We obtained the consent of the Bioethics Committee (number of ethical commission: 9/01/2020) to access the data from the NHF’s central office. One of the co-authors (A.Ś.) was responsible for obtaining the data. Furthermore, we used the data available on the website of Polish Central Statistical Office, which collects and discloses statistical information. We received information on the average length of patients’ life as well as the number and type of services funded by the NHF. Therefore, it was a retrospective study as we did not have complete data but only the processed ones. The data that we obtained were fully anonymised. Because this was an observational retrospective study of fully anonymized patient data collected by a public institution, informed patient consent and ethics committee approval were not required. The results were obtained from February 2016–December 2018.

The patients’ data were reported according to the International Classification of Diseases ICD-10 along with the diagnosis: E84; E84.0; E84.1; E84.8; E84.9—Cystic Fibrosis (CF)—Table S1. All patients had confirmed diagnosis of CF by sweat test ≥60 mmol/L and CFTR gene mutations.

Furthermore, the analysis focused on services financed by the National Health Fund in Poland (third-party public payer of national health insurance), taking into account the frequency of use of outpatient and inpatient medical care and the drug program by CF patients in Poland. We are aware that certain data may be missing, for example due to wrong coding of the cause of death or use of private health services abroad, but we estimate that such data may represent less than 1% of the whole records.

The personal ID number (PESEL) was used as the unique patient’s identifier. The analysis covered the data for which the patient’s ID number (PESEL) and the aforementioned diagnosis symbols existed concurrently. The data were related to all types of healthcare financed by the third-party public payer mentioned in Table 1.

The results were processed using the SAS Enterprise Guide 7.1. and SAS Miner software (SAS Institute, Cary, NC, USA). For the characteristics of the CF population followed between 2000–2018, simple descriptive statistics (mean ± SD, median and proportions) were used. Average death age per year and death rate in paediatric CF patients (deaths per year at paediatric age/total number of recorded patients in that year) were calculated. Normality of distribution was validated using Shapiro–Wilk test, skewness and kurtosis values, as well as based on visual assessment of histograms. Trends over time and differences between groups were evaluated using nonparametric tests (Mann–Whitney U test) and linear regression analysis (Statistica, version 13.1, StatSoft Inc. 2016, Tulsa, OK, USA, www.statsoft.com). All tests were two-tailed with α = 0.05.

## 3. Results

The estimated number of CF patients in Poland is 2400, with approximately 80 CF infants born annually up to 31 December 2018. This is an estimated number as there are no accurate epidemiological data due to lack of a nationwide registry kept for these patients. Moreover, Polish data were often missing in international registries.

**Figure 1:**
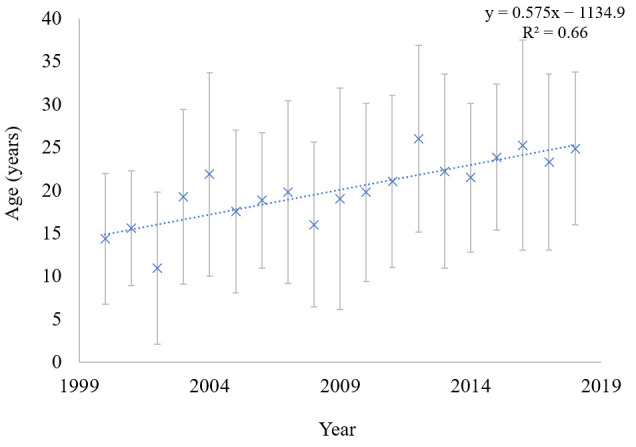
Average life span of cystic fibrosis (CF) patients in the 2000–2018 period.

Figure 1 shows the growing life span of CF patients in Poland during the study period. The average lifetime increased from ca. 14.5 ± 7.6 years (95% CI: 11–17.75 years) in 2000 to 24.5 ± 8.9 years (95% CI: 20.8–28.9 years) in 2018 (mean ± SD, *p* = 0.0001). Variation in the age of patient deaths depends on the age and number of patients who died in a given year. For example, in 2002, 17 patients who were 0–28 years old died, while in 2004, 16 patients who were 6–53 years old died. Similarly, 17 patients died in 2013, the youngest of whom was 13 years old and the oldest 58 years old.

**Figure 2:**
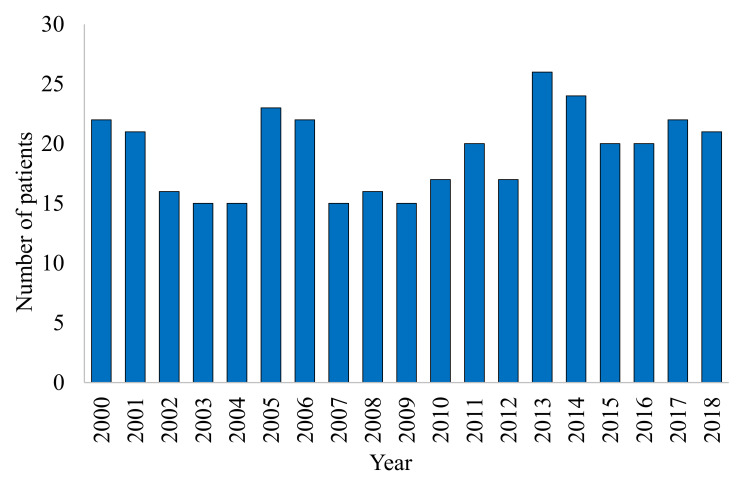
The number of deaths of patients with CF in the 2000–2018 period.

Figure 2 presents the number of CF patients who died between 2000–2018. There were 367 deaths from 2000–2018, at a mean ± SD (range) of 19.3 ± 3.5 (15–26, 95% CI: 17.6–21) deaths/year. We observed no changes in total mortality in the studied period (*p* = 1).

**Figure 3:**
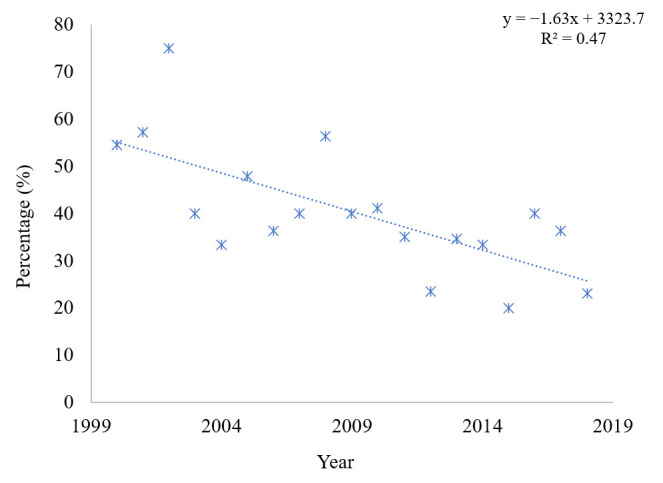
Percentage of deaths of paediatric CF patients in the 2000–2018 period.

Figure 3 shows the percentage of deaths of patients aged 0–18 in relation to all deaths of CF patients in the period from 2000–2018. In the studied period, there were 147 deaths at paediatric age, at a mean ± SD (range) of 7.7 ± 2.6 (4–12, 95% CI: 6.5–8.9) deaths/year. A drop in the death rate can be seen in those patients in the study period: From 55% in 2000 to 22.5% in 2018. Moreover, the mean age of death in paediatric age increased from 8.7 ± 5.1 years in 2000 to 13 ± 4.2 years in 2018.

**Figure 4:**
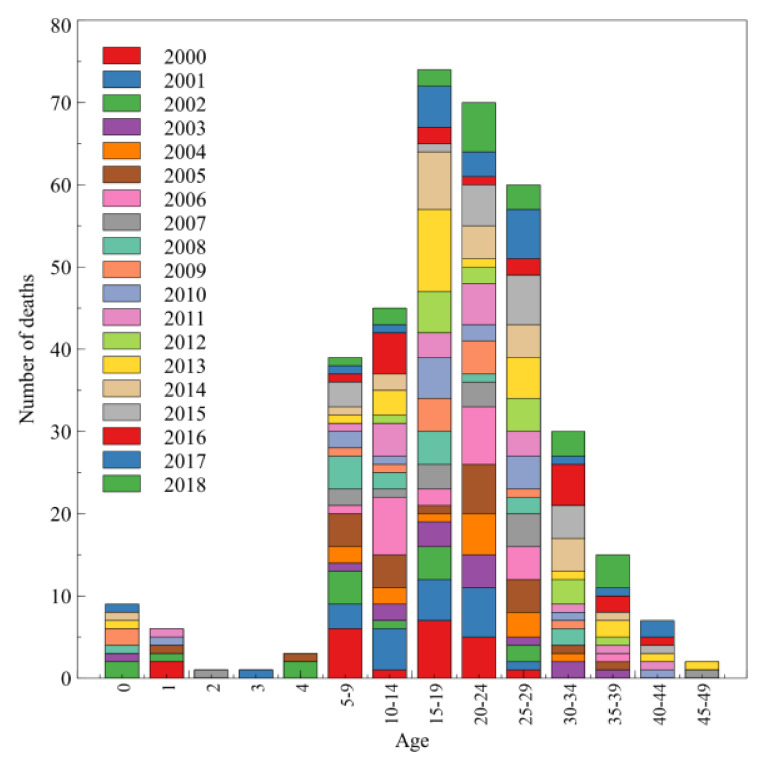
Number of deaths depending on the patient’s age.

Figure 4 shows distribution of the number of deaths depending on the age of the patient, taking into account the period of 2002–2018. The analysis shows that most patients die at 15–24 years of age. During the study period, the lowest mortality was recorded at the age of 2 and 3 years. The highest number of deaths was recorded in 2012 and 2013 when the average age of deaths from CF ranged from 14.5–19.5 years. The lowest number of deaths was recorded in 2007 with 19.5 as the median age at death.

Currently, there are six reference and highly specialised centres in Poland where over 1350 patients (ca. 70% of the CF population) receive care. In each of these centres, the group of patients ranges from 100 to as many as 400. There are also eight smaller centres with 35–80 patients (overall ca. 500 patients, or 25.5% of the CF population). Seven other centres provide treatment to approximately 100 patients (ca. 5% of the CF population), and the number of patients receiving care in other centres ranges from 6–20. Analysis of the services provided in individual provinces (voivodeships) reflects to some extent the model of care and scope of intervention in the reference centres.

The data on the number of patients diagnosed with E84 who used the serviced financed by the National Health Fund during the 2008–2018 period for all categories of outpatient and inpatient care services are presented in Table 2.

Generally, between 2008 and 2018, the number remain constant, with slight deviations observed in the number of patients using the NHF’s services.

During the 2008–2018 period, slight changes in the number of reported patients NHF’s services were observed in all voivodeships; only in the Pomorskie Voivodeship did the number of patients decrease significantly from 617 to 211. In the Małopolskie Voivodeship, on the other hand, the number of CF patients increased from 359 in 2008 to 590 in 2018.

As the estimated population of CF patients in Poland is 2400 and the total number of services reported in 2018 was 3081, it seems that one patient used the services only 1.3 times during the year.

**Figure 5:**
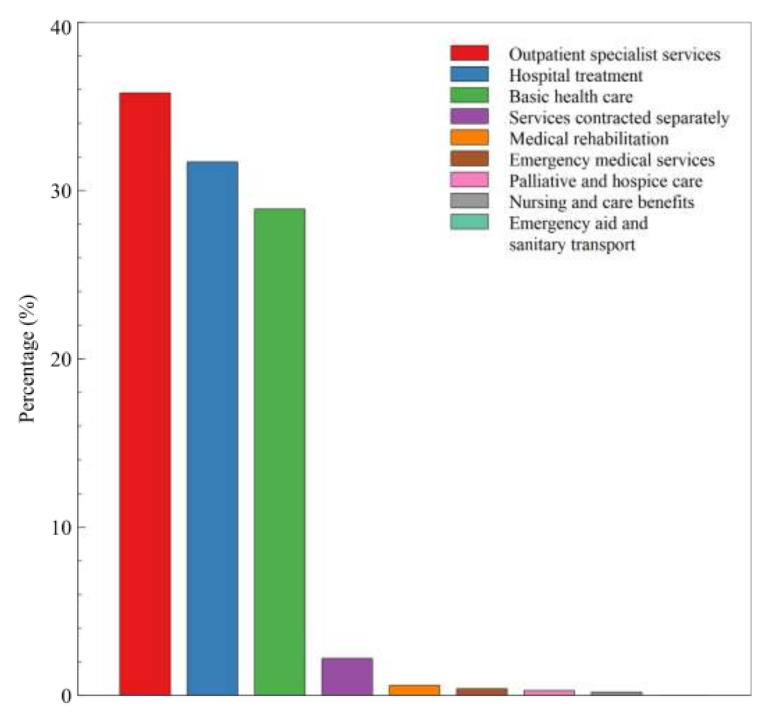
Percentage of patients diagnosed with E84 who benefitted from various services in 2018.

Figure 5 presents healthcare services financed by the National Health Fund used by Polish CF patients in 2018. Most services were provided as part of outpatient specialist care (35.8%), followed by hospital treatment (31.7%) and primary care (28.9%). Other types of benefits are marginal.

**Figure 6:**
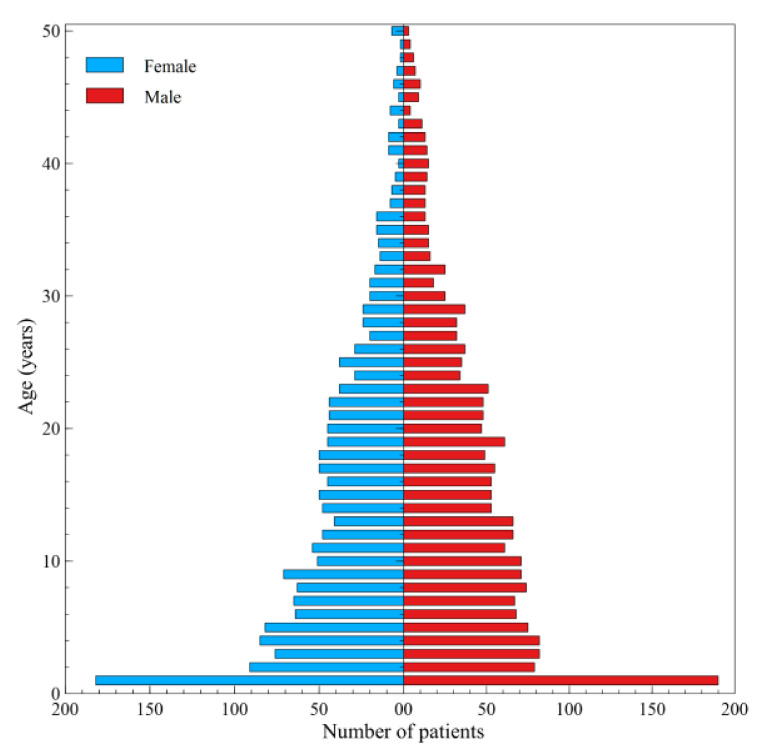
Distribution of services financed by NHF according to patients’ gender and age for all types of services and hospital care in 2018.

Figure 6 presents the number of patients with CF according to gender and age, who benefited from healthcare services in 2018. The largest number of beneficiaries are children under 1 year of age. The number of individuals receiving healthcare services financed by the NHF decreases with the age of patients. There is no significant difference (*p* = 0.27) in the number of men (47.5%) and women (52.1%) who benefit from the services.

Table 3 presents data on the number of patients diagnosed with E84 using services financed by the National Health Fund in 2008–2018 as part of hospital treatment. Generally, in the 2008–2018 period, there was a growing trend in use of hospital treatment by CF patients. Hence, the number of other healthcare services financed by the National Health Fund dropped.

The number of patients with CF receiving hospital treatment increased significantly between 2008–2018 in the Dolnośląskie, Małopolskie, Mazowieckie, and Wielkopolskie Voivodeships. In other analysed provinces, the number of patients stayed at a similar level during the study period.

**Figure 7:**
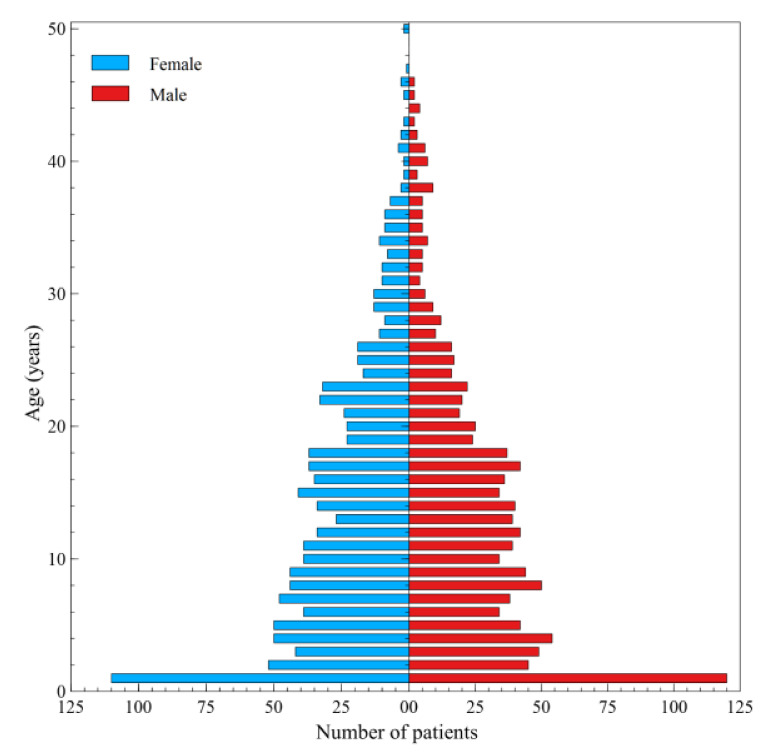
Distribution of hospital services according to the patient age and gender in 2018.

The distribution of hospital treatment financed by the NHF by gender and age is presented in Figure 7. The figure shows that the largest number of patients benefit from the services in the first year of life. In subsequent years of life, there is a systematic decrease in this number. There is no relationship between the use of benefits and gender (50.8% men vs. 49.2% women, *p* = 0.82). As part of hospital treatment, the median age of men was 23 years (mean 25.2) and of women 25 years (mean 28.2).

Table 4 presents data on the number of drug packages—inhaled tobramycin—released in 2009–2017 for patients diagnosed with E84 as part of a drug program financed by the NHF (Treatment of Chronic Lung Infections in Patients with Cystic Fibrosis). Generally, the number of released tobramycin increased from 52 in 2009 to 115 in 2017 over the study period.

**Figure 8:**
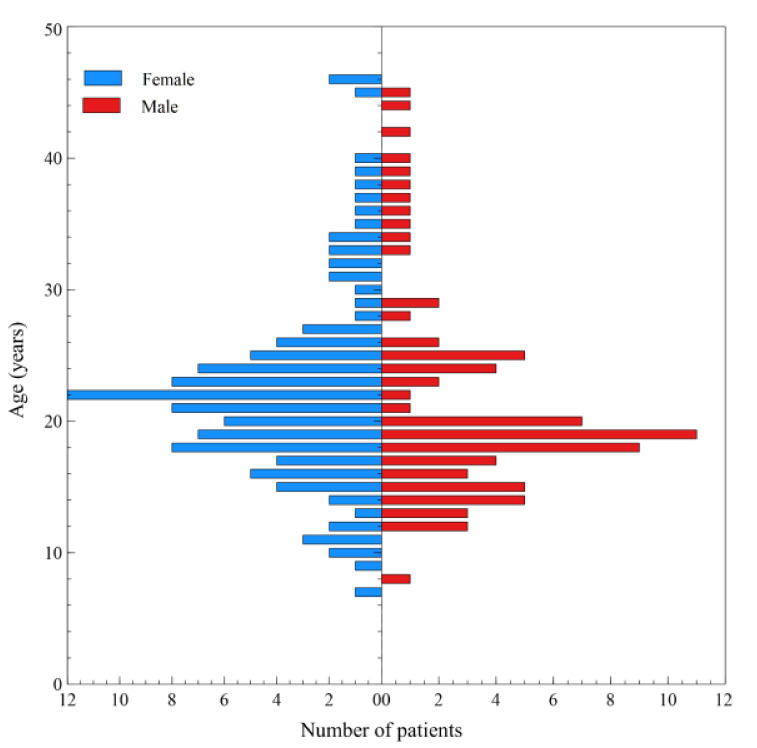
Distribution of drug program services according to the patients’ age and gender in 2017.

Analysis of drug program beneficiaries with CF is presented in Figure 8. It is clear that more female patients (57.1% women vs. 42.9% men, *p* = 0.046) use the drug program of inhaled antibiotic therapy. In the drug program, patients’ median age was 25.5 years for men (mean 25.6) and 25 years for women (mean 25.3). Subsequent conclusions of the analysis showed that these patients are most often aged 15–26 years in the female group and 16–21 years in the male group.

## 4. Discussion

According to the analyses, CF is no longer considered to be a fatal disease of childhood. In the 1950s, the mean length of life of CF patients was only several months. Owing to huge progress in medicine, mainly through improvement of mucus removal from the respiratory tract, management of lung infection, reduction of the symptoms of malabsorption, and pharmacogenetics, the clinical picture of the disease changed. For the last 60 years, the median age of survival has increased and now exceeds 40 years in developed countries, while the mortality has been decreasing [23,24,25].

The median age of patients with CF in years 2000–2018 ranged from 15–25 years. It seems that countries where healthcare seems to be well funded have a much longer life expectancy, as exemplified by Canada. In 2011, the median age of survival in Canada was even 10 years higher than in the United States (50.9 vs. 40.6 years, respectively) [26]. On the other hand, Fischer et al. [27] revealed in 2014 that the median age of CF patients treated in Austrian CF outpatient clinics is only 18.9 years. National cystic fibrosis reports from many highly developed countries, which were published in 2018, indicate that the median age of survival ranges from 44 years in Ireland, 47 in the UK and the US, up to 52 in Canada [28,29,30,31]. Moreover, in Canada, the proportion of adult patients with CF had more than doubled in 35 years, increasing from 30% in 1984 to almost 62% in 2018 [31]. According to the analysis by Keogh et al. [32], it is expected that more than half of children with CF born today will live to at least the fifth decade of life. Access to modern treatment, such as CFTR modulator therapies, contributed to the extension and improvement of patients’ life quality [14].

It is obvious that the patient’s age was correlated significantly and positively with the mean total cost per patient. According to the analysis by Hollmeyer et al. [33], the mean staff cost was €142.3 per CF patient over six months of outpatient service in Germany, while services provided by physicians were the largest contributor to the costs. Moreover, Horvais et al. [34] estimated that the overall cost of CF care totalled €16,189 per year and per patient. The outpatient care represented 88% of the total cost, while 12% was inpatient cost. Chronic medications accounted for almost 50% of total cost, whereas 20% of the cost was spent on home intravenous antibiotic treatments. In the USA, average spending nearly doubled from roughly $67,000 per CF patient in 2010 and 2011 to approximately $131,000 per patient in 2016. During this period, spending on outpatient and inpatient care increased by 0.5% and 2.5% per year, respectively, whereas pharmaceutical spending increased by 20.2% per year [35]. According to the economic analysis by van Gool [36], the majority of costs for treating Australian patients with CF were accounted for by hospital inpatients (58%), followed by pharmaceuticals (29%), medical services (10%), complications (2%), and diagnostic tests (1%). There is no financial analysis of expenses related to cystic fibrosis in Poland.

Unfortunately, no decrease in total mortality over time was observed in Poland in the studied period, which may be due to lack of availability of health services, including drug program, for patients. In England and Wales, the median death age increased from age band 0–4 years to 25–29 years between 1959 and 2008. Moreover, the number of deaths per study year fell during the study period, with a maximus of 214 in 1960 and a minimum of 91 in 2006 [7]. In Spaniards, the average death age for the 36-year period was 27.7 years. In the first part of the study between 1981 and 1998, the average death age was 13.7 years, while in the second part (1999–2016) it was 41.1 years [8]. The European study from 27 countries of the CF population in 1994–2010 revealed that females had a slightly higher mortality rate than males, with a downward trend observed for both genders. The peak of mortality was observed at age 20–24, which is similar to our results. Furthermore, the European study showed an increase in the death age from 17.9–30.3 years for females and from 21.5–29.8 years for males, which is similar to the results observed in Poland [9].

The registry data indicate the percentage of paediatric deaths depending on the country, which ranges 10–20%, and which may reflect the availability of diagnosis and treatment of CF in a given country [6,37].

Ensuring high standards of care requires adequate staff and facilities for the number of patients in need. The cost of care for each patient is lifetime, which increases with the increase of survival, as observed in many countries, including Poland [1,24,38].

In Poland, the distribution of services in individual regions reflects patient access to CF treatment centres. A characteristic local trait is the unequal distribution of patients in centres. In some of the centres, which provide care to groups consisting of several patients, care facilities are insufficient. The highest concentration of patients is noted in Małopolskie and Mazowieckie Voivodeships where there are two largest paediatric centres, each providing care to approximately 400 patients: The Institute of Tuberculosis and Lung Diseases, Regional Department in Rabka-Zdrój in the Małopolskie Voivodeship, and the Institute of Mother and Child in Warsaw, Cystic Fibrosis Centre in Dziekanów Leśny in Mazowieckie Voivodeship, and also the Institute of Tuberculosis and Lung Diseases in Warsaw—the largest centre for sick adults in Poland with about 100 patients. The significant concentration of services in Wielkopolskie Voivodeship is the result of activities of the two important centres covering groups of over 100 patients at the Transfiguration Hospital in Poznań and the Karol Jonscher Clinical Hospital of Karol Marcinkowski Medical University in Poznań. In 2018, the number of services provided in Mazowieckie Voivodeship increased due to transferring the then existing paediatric centre of the Institute of Mother and Child to the Dziekanów Leśny facilities. The year 2017 was the first year—and a very busy one—of this modern medical centre, now a model example of a high-end CF treatment facility; therefore, it may be assumed that increase in services provided in the Mazowieckie region is an effect of the centre’s activity. Generally, in recent years, Poland has witnessed an increase in number of centres providing CF treatment. In 2011, there were 10 such centres, and only in 4 of them the number of patients exceeded 50 per centre [39]. Now the patients are treated in 27 centres, and 14 of them provide treatment to more than 35 patients each. Currently, only nine centres in Poland report data to the ECFS Patient Registry. In the 2017 report, only 721 patients were registered, including 79.8% of children and 20.3% of adults. Countries with national registers, such as France or Germany, have more than 80% of patients registered in the report [27].

A significant decrease in number of patients in Pomorskie Voivodeship is related to the migration of patients to other health centres, especially to the Cystic Fibrosis Centre in Dziekanów Leśny. There is only one CF treatment centre in Northern Poland—the Cystic Fibrosis Outpatient Treatment Centre in Gdańsk. The lower number of patients in the study period in many of the centres may result from CF patient migration to centres with a higher reference level, or from technical errors in coding and lack of regular reporting to the NHF. Between 2000 and 2018, smaller pulmonary outpatient clinics were established closer to patients’ place of residence. These clinics took over some of the patients from larger health centres.

The presented analysis shows that CF patients in Poland use specialist healthcare services to a negligible extent. This may be a result of lack of ICD10-compliant coding or imprecise coding by doctors, as well as from limited access to specialists. It is believed that this may also result from transfer of some of the patients to general practitioners’ care, and ultimately lack of awareness of carers/patients that they may take advantage of specialist services. In addition, a number of patients may use private healthcare, which is not subject to reporting. We estimate that less than 1% of patients use private health services, including health services in other countries (Sweden, Austria, and Germany).

Services provided as part of specialist outpatient care represent the highest number of services financed by the NHF; the next in line are inpatient care services and primary care. The large number of specialist outpatient and inpatient services provided to CF patients results from controlling the course and treatment of the underlying disease. It seems that treatment in larger and multidisciplinary centres will contribute to better patient outcomes. However, according to the analysis by Post et al. [40], outcomes of CF patients were not better in specialised centres or with subspecialists compared to other forms of chronic illness care, including primary care generalists.

The highest number of patients use the services in the first year of life. This is a result of hospitalisation of infants who had tested positive in CF screening tests. Furthermore, the children tend to undergo detailed diagnostic examinations and their parents/carers receive guidelines as to their diet, daily care, and physical therapy. Unfortunately, despite recommendations concerning physical therapy, CF patients practically do not use these type of services [41]. Current rules of providing specialist outpatient or inpatient care services do not put an obligation on CF treatment centres to provide physiotherapeutic services. As in other countries, one must note poor access to physiotherapists experienced in physical therapy of CF patients at the patients’ area of residence [42,43].

An example of drastic and medically unreasonable restrictions on access to modern and effective technologies is the tobramycin program “Treatment of Chronic Lung Infections in Patients with Cystic Fibrosis”. The drug is the antibiotic of choice for the treatment of CF patients with chronic respiratory infection caused by blue oil rod (*P. aeruginosa*). It should be emphasised that tobramycin preparations are reimbursed without restrictions in most European countries. Unfortunately, the criteria for including patients in the program in Poland are very strict, which results in low access to the treatment. Under the program, tobramycin is available to the patient only in case of bacterial resistance to the previously administered drug (colistin) or if that drug is not tolerated by the patient. It is estimated that this may affect ca. 10–15% patients; therefore, taking into account the aforementioned criteria, only such a small percentage of patients may benefit from the therapy. Inhaled tobramycin not only improves lung function in patients with CF, but also offers other benefits that have impact on the healthcare cost and the patient’s quality of life [44,45]. Benefits such as improved patient nutrition and reduced need for hospitalisation have been documented [46]. Organisational difficulties and restrictive criteria for inclusion in the program mean that reimbursed tobramycin is used only in a small group of patients (currently a dozen or so people). This is clearly demonstrated by the treatment results of Polish CF patients. The drug remains unavailable to a large number of patients who might benefit from its use. Considering the recommendations for the routine use of this antibiotic, it is necessary to broaden access to this drug by changing the inclusion criteria or adding the drug to the reimbursed drugs list. According to the standard, all patients in Poland benefit from basic treatment financed by the NHF, which includes hypertonic saline, Pulmozyme (dornase alfa), pancreatic enzyme replacement therapy, and vitamins. It is worth noting that the highly effective CFTR modulator therapy is not widely available in Poland. As these preparations were used only in clinical trials and in one drug program, their availability was very limited. For many years, efforts have been made to adjust the Polish patient treatment system to European standards. At the same time, Polish patients cannot benefit from consultations of multidisciplinary teams due to lack of proper valuation of cystic fibrosis health services by the National Health Fund. There is a lack of systemic solutions that would provide comprehensive and coordinated care for patients with cystic fibrosis. The most important changes take place in large centres; unfortunately, the treatment received by CF patients continues to deviate from the European, Canadian, or American standards and this has impact on the presented results. We should mention that the medical staff caring for the patients in Poland is a unique group of enthusiasts dedicated to their work and striving to do their best within the limited resource available.

We believe that there is no adequate valuation of outpatient services by the National Health Fund that would allow for the provision of multidisciplinary outpatient consultations. However, there are also other reasons for the difference, which may include a small number of multidisciplinary centres and patients’ limited access to highly specialised services or personnel qualified for treatment of rare diseases (e.g., physiotherapists, nutritionists, and social workers). In addition, we believe that the reason may also be the low number of home treatment programs (physiotherapy, antibiotic therapy), lack of modern drugs, lack of psychosocial support for patients and their families, and the persistently low number of transplantations. A substantial number of medications and special dietary products available to patients are not reimbursed under the healthcare insurance, which is a large financial burden for patients. Finally, due to the lack of adequate infrastructure, patients cannot be adequately isolated in health centres, which leads to cross-contamination and, consequently, deterioration of their health. Generally, compared to other countries, Poland seems to lack systemic and financial solutions to ensure comprehensive care for patients.

In summary, in this study, we collected the data on mortality and patient use of NHF-funded services from the population attributable to CF in the period from 2000 to 2018. Despite the increase in life expectancy and reduction of child mortality, the use of health resources, especially drug programs, in patients with cystic fibrosis is low. There are no data confirming the impact of access to the highly specialist centres focusing on CF treatment on the number of deaths. The limitation of the study is the lack of demographic and clinical characteristics of the patients (e.g., pulmonary function, pancreatic sufficiency, number of exacerbations per year, CFTR genotype) that could be related to some outcomes of this study. Moreover, socioeconomic status was not taken into account in this article because the noted results were not consistent in all regions of the country.

In future, development of health policy will have to consider the possibility of regional differentiation due to the availability of treatment. Moreover, the requirements are directed at expanding into multidisciplinary teams, mutual isolation of patients, introducing a comprehensive and coordinated care network with precise division of tasks between system participants, strengthening home care, providing access to food for special dietary purposes, and optimal drug therapies.

## 5. Conclusions

Despite the observed progress of extending life expectancy and improving the quality of care for patients with CF in Poland, many aspects of health care still differ from the treatment standards in other developed countries.

The key element determining the improvement of the patients’ health system is the introduction of such systemic and financial solutions that will guarantee patients access to comprehensive, modern, and multidisciplinary therapy in line with European standards. It seems that implementation of the new solution in form of organizational changes, identifying this comparatively rare but serious disease as a separate disease entity, and finally ensuring proper funds from NHF for services dedicated to patients with CF, will allow for better use of the opportunities available in medical care, including access to the drug program. In addition, the key seems to be to increase the expenditure on modern therapies that will significantly improve the quality of life of patients.

## Figures and Tables

**Table 1:** Types of services covered by the National Health Fund (NHF) in Poland.

Healthcare Services	
01	Basic health care
02	Ambulatory specialist benefits
03	Hospital treatment
04	Psychiatric care and addictive treatment
05	Medical rehabilitation
06	Long-term care
07	Dental treatment
09	Advisory assistance and sanitary transport
10	Preventive health programs
11	Independent contracting benefits
12	Care and care
13	Palliative and hospitable care
14	Medical rescue
15	Advisory assistance and sanitary transport

**Table 2:** Number of patients by voivodeships for all types of services.

Voivodeship	2008	2009	2010	2011	2012	2013	2014	2015	2016	2017	2018
Dolnośląskie	180	173	176	170	172	190	183	201	208	227	190
Kujawsko-Pomorskie	131	153	123	129	126	118	133	137	157	151	117
Lubelskie	82	92	100	91	93	96	97	94	80	90	47
Lubuskie	60	85	70	58	53	53	54	46	51	55	42
Łódzkie	164	177	220	268	303	343	303	221	197	173	163
Małopolskie	359	433	483	466	486	533	523	526	572	577	590
Mazowieckie	617	675	673	666	660	667	633	624	590	740	710
Opolskie	74	68	68	58	63	57	61	60	60	52	50
Podkarpackie	85	103	105	129	138	126	107	109	116	113	92
Podlaskie	79	92	83	97	98	109	92	78	86	70	57
Pomorskie	617	738	675	628	523	553	251	234	234	226	211
Śląskie	263	373	383	384	355	397	377	366	363	345	325
Świętokrzyskie	49	69	65	60	62	71	73	65	69	66	50
Warmińsko-Mazurskie	46	50	42	44	58	51	48	56	56	53	41
Wielkopolskie	239	298	271	287	307	323	335	331	356	363	320
Zachodniopomorskie	70	71	84	102	102	102	100	108	102	121	76
TOTAL	3115	3650	3621	3637	3599	3789	3370	3256	3297	3422	3081

**Table 3:** Number of E84 patients using hospital services financed by the National Health Fund during the 2008–2018 period.

Voivodeship	2008	2009	2010	2011	2012	2013	2014	2015	2016	2017	2018
Dolnośląskie	55	61	55	77	98	97	108	116	139	139	123
Kujawsko-Pomorskie	58	38	39	28	38	33	35	35	44	48	37
Lubelskie	24	30	22	25	25	23	22	28	22	20	21
Lubuskie	4	5	5	8	4	5	5	7	6	8	6
Łódzkie	40	36	43	34	47	43	60	52	37	47	33
Małopolskie	313	330	393	369	413	468	454	456	487	494	510
Mazowieckie	306	295	415	406	357	400	384	389	311	503	690
Opolskie	12	6	2	7	3	5	4	6	5	8	4
Podkarpackie	26	27	18	23	32	27	17	34	32	40	51
Podlaskie	23	22	19	22	16	19	18	22	26	31	20
Pomorskie	108	124	121	114	108	120	125	116	87	95	82
Śląskie	56	56	53	50	62	46	46	47	66	83	74
Świętokrzyskie	7	18	15	15	18	21	21	18	21	22	19
Warmińsko-Mazurskie	6	9	4	4	12	8	8	7	6	4	4
Wielkopolskie	145	173	197	204	215	205	223	221	238	234	210
Zachodniopomorskie	28	31	25	37	31	35	26	24	25	37	24
TOTAL	1211	1261	1426	1423	1479	1555	1556	1578	1552	1813	1908

**Table 4:** Number of drug packages released to E84 patients during a year as part of the program “Treatment of Chronic Lung Infections in Patient with Cystic Fibrosis” *.

Voivodeship	2008	2009	2010	2011	2012	2013	2014	2015	2016	2017	2018
Dolnośląskie	n/a	7	12	10	13	17	16	16	18	17	n/a
Kujawsko-Pomorskie	n/a	n/a	4	4	4	4	4	4	5	5	n/a
Lubelskie	n/a	n/a	1	1	3	1	1	7	9	6	n/a
Lubuskie	n/a	4	5	9	9	7	6	7	8	0	n/a
Łódzkie	n/a	14	17	10	7	7	9	10	12	8	n/a
Małopolskie	n/a	5	7	6	8	5	9	19	22	15	n/a
Mazowieckie	n/a	3	3	4	3	6	3	2	1	22	n/a
Opolskie	n/a	3	2	1	4	4	4	6	7	0	n/a
Podkarpackie	n/a	5	5	4	5	7	7	5	5	0	n/a
Podlaskie	n/a	4	5	5	5	7	8	8	8	9	n/a
Pomorskie	n/a	n/a	4	5	6	7	7	8	7	7	n/a
Śląskie	n/a	n/a	1	2	6	9	12	10	9	8	n/a
Świętokrzyskie	n/a	n/a	n/a	n/a	n/a	n/a	1	1	1	5	n/a
Warmińsko-Mazurskie	n/a	7	12	10	13	17	16	16	18	n/a	n/a
Wielkopolskie	n/a	n/a	4	4	4	4	4	4	5	12	n/a
Zachodniopomorskie	n/a	n/a	1	1	3	1	1	7	9	1	n/a
TOTAL	n/a	52	83	76	93	103	108	130	144	115	n/a

Note: *—n/a—data no available.

## Supplementary Materials

The following are available online at https://www.mdpi.com/1660-4601/17/20/7630/s1, Table S1: Inclusion/exclusion criteria according to International Classification of Diseases, Ninth Revision (ICD-9) for procedures and International Classification of Diseases, Tenth Revision (ICD-10) for diagnosis.
