# Supplementary material for: Managing Cystic Fibrosis in Polish Healthcare
Source: Int J Environ Res Public Health. 2020 Oct 20;17(20):7630. doi: 10.3390/ijerph17207630 (PMC7589707; doi:10.3390/ijerph17207630)
Supplement: Supplementary file 1 [file ijerph-17-07630-s001.pdf]

Table S1. Inclusion/exclusion criteria according to International Classification of Diseases, Ninth Revision (ICD-9) for procedures and International Classification of Diseases, Tenth Revision (ICD-10) for diagnosis.

Cystic fibrosis with pulmonary manifestations	E84.0
Cystic fibrosis with intestinal manifestations	E84.1
Cystic fibrosis with other manifestations	E84.8
Cystic fibrosis, unspecified	E84.9
